# Sequencing Effects of Concurrent Strength and Endurance Training on Selected Measures of Physical Fitness in Young Male Soccer Players: A Randomized Matched-Pairs Trial

**DOI:** 10.1186/s40798-024-00726-4

**Published:** 2024-05-23

**Authors:** Roland Blechschmied, Matthijs Hermse, Martijn Gäbler, Marije Elferink-Gemser, Tibor Hortobágyi, Urs Granacher

**Affiliations:** 1 Department of Sport and Sport Science, Exercise and Human Movement Science, University of Freiburg, Sandfangweg 4, 79102 Freiburg, Germany; 2 Department of Human Movement Sciences, University Medical Center Groningen, University of Groningen, Groningen, Netherlands; 3 Department of Kinesiology, Hungarian University of Sports Science, Budapest, 1123 Hungary; 4 Department of Sport Biology, Institute of Sport Sciences and Physical Education, University of Pécs, Pécs, 7622 Hungary; 5 Department of Neurology, Somogy County Kaposi Mór Teaching Hospital, Kaposvár, 7400 Hungary; 6 Institute of Sport Research, Sports University of Tirana, Tirana, Albania

**Keywords:** Adolescent, Cardiorespiratory Fitness, Muscle Strength, Resistance Training, Youth Sports

## Abstract

**Background:**

Various physical fitness qualities such as muscle strength, speed and endurance are related to soccer performance. Accordingly, the combination of strength and endurance training (i.e., concurrent training [CT]) is an often-encountered training regimen in soccer. Less is known about the effects of CT sequencing on performance in young soccer players. The aim of this study was to assess the sequencing effects of strength and intermittent endurance training applied within the same training session (intrasession) on measures of physical fitness and soccer performance in young soccer players.

**Methods:**

Fifty male adolescent soccer players volunteered to participate in this study which was conducted in the Netherlands in 2019. Players were randomly assigned to a strength-endurance (SE) or an endurance-strength (ES) group in matched pairs based on their countermovement jump (CMJ) performance at baseline. Both groups completed a 12-weeks in-season training program with two weekly CT sessions. Training sessions consisted of 15 min plyometric exercises and 15 min soccer-specific intermittent endurance training. Both groups performed the same training volumes and the only difference between the groups was the CT intrasession sequencing scheme (SE vs. ES). Pre and post intervention, proxies of muscle power (CMJ, squat jump [SJ]), linear sprint speed (30-m sprint test), agility (Illinois test with / without ball), and soccer performance (ball kicking velocity) were tested.

**Results:**

Data from 38 players aged 14.8 ± 1.0 years (body height 172.9 ± 8.1 cm, body mass: 57.0 ± 7.2 kg, soccer experience: 8.8 ± 2.8 years, age from peak-height-velocity [PHV]: +1.2 ± 1.0 years) were included. Significant main time effects were found for CMJ (*p* = 0.002, d = 0.55), SJ (*p* = 0.004, d = 0.51), the Illinois agility test with ball (*p* = 0.016, d = 0.51), and ball kicking velocity (*p* = 0.016, d = 0.51). Significant group-by-time interactions were observed for 30-m linear sprint speed (*p* < 0.001, d = 0.76) with ES showing greater improvements (*p* = 0.006, d = 0.85, Δ-5%).

**Conclusions:**

Both CT-sequencing types improved performance in the tests administered. The intrasession CT sequencing (SE vs. ES) appears not to have a major impact on physical fitness adaptations, except for linear sprint speed which was in favor of ES.

**Supplementary Information:**

The online version contains supplementary material available at 10.1186/s40798-024-00726-4.

## Key points

• This study examined the effects of intrasession concurrent training. Findings showed that strength-endurance and endurance-strength sequencing resulted in similar changes in most measures of physical fitness in adolescent male soccer players.

• If the goal is to improve linear sprint speed, endurance-strength sequencing appears to be better suited.

• More research is needed to acquire further knowledge on concurrent training and effects of different types of sequencing schemes (e.g., training day, microcycle) as well as the strength training (e.g., power training) and endurance training type (e.g., high intensity interval training).

**Supplementary Information:**

The online version contains supplementary material available at 10.1186/s40798-024-00726-4.

## Background

Soccer coaches and players are often confronted with the challenge to train aerobic capacity and muscle strength or power within microcycles, on the same training day, or even within the same training session (intrasession) [1, 2]. This concept is called “concurrent training” (CT). While endurance training aims to improve cardiovascular fitness and targets physiological adaptations such as mitochondrial biogenesis and optimizing oxygen uptake [3–5], strength training (e.g., weightlifting, plyometric training) increases muscle strength, power or local muscular endurance by inducing neural (e.g., motor unit recruitment) and/or morphological adaptations (cross-sectional area) [1, 6]. The preferred type of the applied strength training depends on the athlete’s needs in a specific sport. For soccer players, plyometric training displays an often-selected exercise modality, as it supports jumping, landing and change-of-direction (CoD) actions, is easy-to-administer even on the pitch and affords hardly any exercise equipment. Moreover, previous studies have shown plyometric training related gains in muscle power (e.g., countermovement jump [CMJ]), CoD (e.g., 10-m agility test), and linear sprint speed (e.g., 10-m sprint time) in young soccer players aged 13.2 ± 0.6 years [7]. There is evidence that plyometric training has the potential to induce muscle hypertrophy in healthy individuals, irrespective of age, sex or training status [8] and improve measures of muscle strength in non-athletic adolescents aged 16.9 ± 0.8 years (e.g., 5-RM squat) [9], irrespective of the players’ expertise status [10]. Limited information is available in the scientific literature on the combined effects of plyometric and endurance training in youth soccer.

The effectiveness of different CT modalities on measures of physical fitness has been extensively studied in the past and produced conflicting results [11, 12]. Some studies suggest that the concurrent training of strength and aerobic capacity may interfere with physiological adaptations, i.e., preventing the development of the full adaptive potential [13, 14]. This interference effect is often attributed to incompatible intracellular signaling mechanisms between the mechanistic target of rapamycin (mTOR) as the main mediator for strength and hypertrophy training adaptations and AMP-activated protein kinase (AMPK) for mitochondrial biogenesis following endurance training [5, 13]. This phenomenon has been observed in various populations, including prepubescent children aged 10.9 ± 0.5 years [15, 16], young male adults aged 23 ± 0.6 years [17], untrained middle-aged males aged 42.0 ± 2.0 years [5, 18], and recreationally trained male endurance runners aged 23.0 ± 2.0 years [19].

Other studies contradict these interference effects by showing no negative effects of CT compared to single mode strength training on muscle hypertrophy and maximal strength, irrespective of the training status and age (< 40 / > 40 years) [15, 16, 20]. For instance, Wong and colleagues (2010) examined the effects of CT compared to soccer training only on measures of physical fitness in young adult soccer players. CT resulted in larger performance improvements compared with soccer training for the one repetition maximum (1-RM) half back squat and bench press, vertical jump height, 10-m sprint, Yo-Yo-test and the maximal aerobic speed test [21].

More recent studies reported larger training effects following CT compared to single-mode endurance or strength training on aerobic capacity, exercise economy, and time trial performance in young athletes aged 10–18 years [11]. As the previously outlined research indicates, there are large discrepancies regarding the effects of CT on measures of physical fitness in youth soccer players. These variations can be attributed to several factors, including discrepancies in age, biological maturation, and training status of the studied cohorts, each of which may exhibit unique responses to CT interventions. Moreover, the mentioned distinct CT modalities (microcycle, same day, intrasession) seem to further present an influential factor for subsequent training-induced adaptations. Lastly, the included strength (e.g., weightlifting, plyometric training) and endurance (e.g., high-intensity-interval, aerobic) training modalities might introduce an additional moderating factor for induced training adaptations. As a result, future research should take these moderators into account when designing CT studies in youth.

Due to the limited time for training and the intermittent metabolic demands of soccer, CT has become an often applied exercise regimen in youth soccer [11]. Although many studies have been published on the effects of CT vs. single mode strength or endurance training on physical fitness in youth [11, 12], less is known on the intrasession sequencing of concurrent strength and endurance training (e.g., strength-endurance [SE] or endurance-strength [ES]). The aggregated current evidence suggests distinct levels of adaptation for different age and maturity groups following the two opposite sequencing schemes [7, 8].

In summary, adolescent males aged 14 to 17 show different training adaptations relative to the applied sequencing schemes (SE vs. ES) and these adaptations seem to further depend on their maturity status. Generally, mature youth athletes (post-peak height velocity [PHV]) seem to be more prone to an interference effect in the form of blunted muscle hypertrophy and strength gains when endurance training was performed after strength training [11, 12].

This is likely a result of higher adaptive potential for morphological responses such as muscle hypertrophy due to increased levels of androgenic hormones (i.e., testosterone) in pubescent and postpubescent youth [11]. Higher potential for morphological adaptations also implicate a greater chance of experiencing interference effects induced by the mTOR and AMPK pathway mechanisms [5, 13].

Therefore, considering the available scientific literature [11, 12], we hypothesized that the sequencing scheme ES would provide greater adaptive potential than SE on selected measures of physical fitness (e.g., muscle power, jumping performance, speed) and soccer performance (e.g. ball kicking velocity) in adolescent soccer players. Accordingly, the purpose of the current study was to examine the effects of intrasession CT sequencing (SE vs. ES) on physical fitness and soccer performance in adolescent male soccer players.

## Methods

To test our hypothesis, adaptations following intrasession SE or ES were analyzed using a randomized matched-pairs intervention study design. Players were ranked and matched in pairs based on their CMJ performance at baseline. Using these data, they were randomly assigned to one of the intervention groups. The aim was to ensure an even distribution of lower limb muscular performance at baseline.

### Participants

**Fig. 1:**
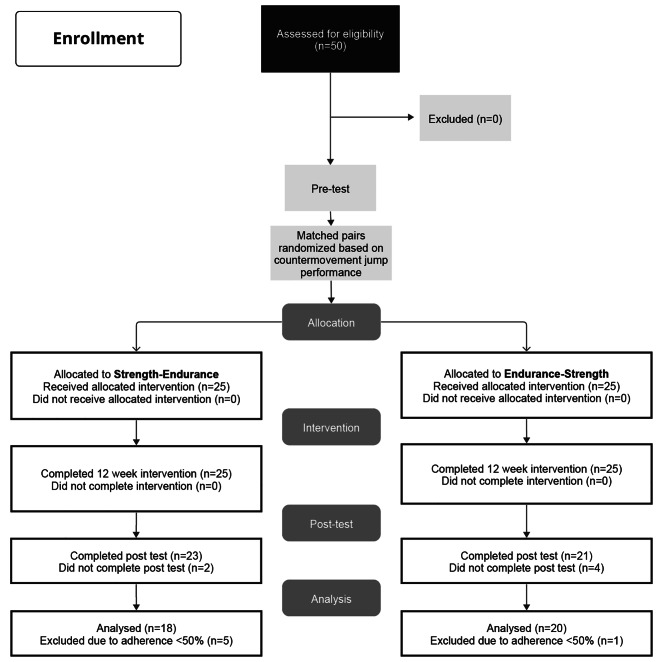
Flow chart of the progress through the phases of the study according to the CONSORT statements

Before the start of the study, 50 injury free male young competitive elite soccer players (elite score 4.6 in elite scale; range: 1–4 semi-elite; 4–8 competitive elite; 8–12 successful elite; 12–16 world-class elite [22]) competing at the highest regional level of the northern Netherlands [23] volunteered to participate. The required sample size was determined using an a priori power analysis and a related study [2]. The reported effect size for 30-m linear sprint speed (eta^2^ = 0.13) was used and converted into Cohen’s f (f = 0.387) and then computed with an alpha error probability of 0.01 and power of 0.9. The minimum required sample size was determined to be *N* = 30. As we expected drop-outs due to injury, illness (season and weather induced) as well as difficulties with training adherence, we opted for *N* = 50. Players were randomly assigned to SE (*n* = 25) or ES (*n* = 25) groups in matched pairs based on their CMJ height at baseline in an attempt to match groups with regards to their lower body muscle power performance. Inclusion criteria were > 5 y of soccer experience, absence of injury for at least six months prior to the start of the study, age 13–16 years, and highest regional soccer performance level. The participating players were experienced in terms of the regular performance of on-field strength and plyometric exercises as typically used in soccer training (e.g., push-ups, squats). None of the participants had previously performed any systematic strength training in the gym. Players who missed the post-tests or attended less than 50% of the scheduled exercise sessions were excluded from the final data analysis (Fig. 1.). All participants and their legal representatives were informed about potential benefits and risks of the study prior to study participation. Written informed consent was then obtained from the players and their legal representatives. The study was approved by the ethics committee of the Department for Human Movement Sciences, Groningen, The Netherlands (approval number: METc 2018/201,800,807).

### Study Design

The experimental groups completed 12 weeks of CT training and one match on the weekend. CT was applied during two weekly training sessions. The third session comprised of a soccer-specific exercise protocol. The study was carried out during the in-season from February to June 2019. Testing sessions replaced a training session and were held at the very beginning and end of the training period in March and May, respectively. At least 48 h of rest were assured before each testing session. All tests were performed by the same assessor. The test instructions and cues were standardized across pre/post tests. Tests were performed in a circuit with the same test order during pre and post-tests.

#### Proxies for Muscle Power

As proxies for muscle power, CMJ and SJ tests were applied. The tests were performed with hands akimbo (no arm support) using the OptoJump Next® system (Microgate, Mahopac, NY, USA). Jump height was calculated by measuring the flight time using the formula: jump height = 1/8 × g × t², where g is the acceleration due to gravity and t is the flight time [24]. Previously, excellent intra-class correlation coefficients (ICCs) were reported for both tests and amounted to 0.93 (CMJ) and 0.97 (SJ) [25, 26].

#### Speed

Speed was tested using a 30-meter linear sprint test (30-m sprint) and photocell timing gates (TAG Heuer, La Chaux-de-fonds, Switzerland). This method has excellent test-retest reliability with an ICC = 0.94 [27].

#### Agility

Agility was assessed with and without dribbling a ball using the Illinois agility test [28]. The time taken to complete the test was measured using photocell gates (TAG Heuer, La Chaux-de-fonds, Switzerland). The Illinois agility tests has shown excellent test-retest reliability without the ball, ICC = 0.90 [28] and with the ball, ICC = 0.98 [29].

#### Soccer Performance

To assess a specific aspect of soccer performance, ball kicking velocity was measured in km/h using a radar gun (Stalker sport 2, Plano, Texas, USA). For this purpose, participants were asked to shoot the ball at maximal effort from a distance of 11 m into an empty goal. Participants had two trials and if better performance was achieved in trial two, another trial was granted. The best trial in terms of ball kicking velocity was considered for further analysis. Test-retest reliability was excellent for this test with an ICC = 0.94 [30].

### Exercise Protocols

Overall, 36 exercise sessions including 24 CT sessions were applied across the intervention period. The total training volume was similar between the two experimental groups. The applied training protocols were similar to those of Makhlouf et al. [2] as well as Enright and Morton et al. [1].

After a 10-min warm-up consisting of light jogging, dynamic stretching, and low intensity plyometrics, both training groups completed 30 min intrasession CT. The SE group performed 15 min of plyometric training before 15 min of intermittent endurance training (i.e., small-sided games). The ES group performed 15 min of small-sided games prior to 15 min of plyometric training. A short break of 3–5 min was granted before the athletes transitioned from SE to ES and vice versa. Plyometric exercises primarily included CMJs, drop jumps, and lunges.

During training, the following exercise instructions were provided from coaches to athletes: “Perform the jumps at maximal effort and jump as high as possible” [2, 7]. The same trunk muscle strength exercises were applied in both groups (e.g., partner ball toss crunch, side plank crunch) as part of the strength / plyometric training. Small-sided games were conducted on the pitch in a 20-m x 20-m square [1]. For the assessment of exercise intensity during small-sided games, heart rate monitors (Polar, Kempele, Finland) were used. Over the course of the study, intensity during small-sided games was above 80% of individual maximum heart rate. The heart rate maximum was assessed using a 20-m shuttle run test at baseline. Supplement 1 provides detailed information on the programming of the two exercise protocols. After 30 min of CT, 60 min of regular soccer training were scheduled. Regular soccer training involved exercise drills such as shooting towards the goal, two vs. two dribbling and passing drills, and matches on small and large soccer fields. The third weekly exercise session focused on regular soccer training only (no CT) including technical and tactical drills. Here, participants exercised specific tactical maneuvers and strategies in standard situations such as free kicks or corners.

### Statistical Analyses

Normal distribution of data and homoscedasticity were tested and confirmed using the Kolmogorov-Smirnov Test [31] and the Levene test [32]. Independent t-tests were applied to identify possible baseline differences between groups. A mixed ANOVA (within subject factor time: pre/ post, between subject factor groups: ES / SE) was performed to determine main effects of group and time and group-by-time interactions. In case of significant group-by-time interactions, Bonferroni adjusted post-hoc tests were computed. Effects sizes (eta-squared) were computed and transformed into Cohen’s *d* using the following formula: *d* = √(η² * (1 - η²)) * (1 / √2) [33]. Within group Cohen’s *d* was calculated using the following equation: *d* = mean*pre* - mean*post*/SD*pre* [34, 35]. Cohen’s *d* can be interpreted as: < 0.5 = small effect, 0.5–0.8 = medium effect and > 0.8 = large effect [36]. The level of statistical significance was set at *p* < 0.05. All analyses were computed using Statistical Package for Social Sciences (SPSS) version 29 (SPSS inc., Chicago, Illinois, USA).

## Results

### Training

All players received interventions as allocated. Fifty young male soccer players originally met the inclusion criteria. Twelve drop-outs occurred over the course of the study, six due to the absence from post-tests and six because of a training adherence rate below 50% (Fig. 1). Table 1 shows the characteristics of the athletes who completed at least a minimum of 50% of the intervention CT training sessions. Mean adherence for all participants was 68 ± 12%. The final data set comprised 18 players in the SE group and 20 players in the ES group (Table 1). There were no significant between-group baseline differences for any of the assessed outcome variables (Table 1). No training- or test-related injuries occurred during the study.

**Table 1:** Baseline characteristics of the study participants according to group allocation. Values are means ± standard deviations (SDs)

Variable	SE (*n* = 18)	ES (*n* = 20)	*P*
Age (y)	14.9 ± 1.0	14.8 ± 1.0	0.98
Body height (cm)	173 ± 8.5	172.7 ± 7.8	0.91
Body mass (kg)	58.9 ± 8.5	55.5 ± 5.8	0.27
Soccer experience (y)	8.4 ± 4.0	9.2 ± 1.5	0.53
Age from peak height velocity (y)	1.2 ± 1.2	1.2 ± 0.9	0.98
Age at peak height velocity (y)	14 ± 0.5	14.1 ± 0.7	0.73
Training adherence (%)	69 ± 14	66 ± 11	0.48

Abbreviations: 1SE = Strength-Endurance, ES = Endurance-Strength, y = years, cm = centimetres, kg = kilograms

### Measures of Physical Fitness and Soccer-related Performance

**Fig. 2:**
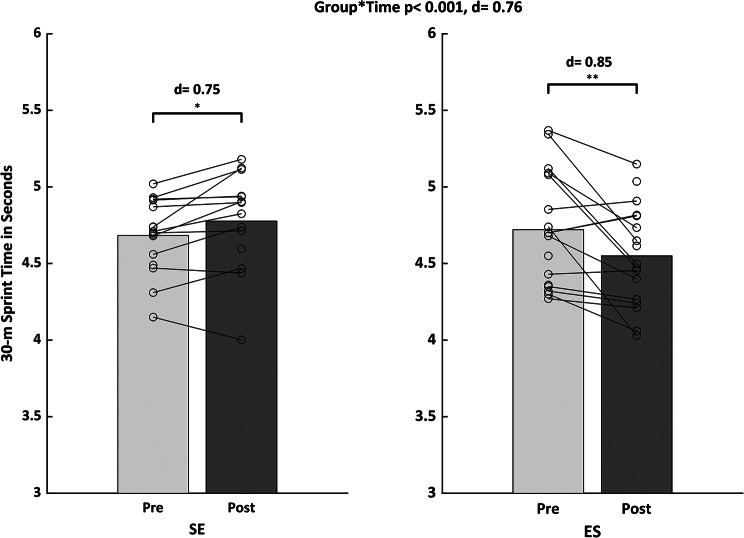
Mean values (bars) and individual scores (lines) for the 30-m linear sprint test Legend: d, Cohen’s d; SE = strength training before endurance training; ES = endurance training before strength training * = *p* < 0.05; ** = *p* < 0.005

Table 2 presents pre and post data for all measures of physical fitness and soccer performance. The ANOVA analysis (Table 2) revealed significant main effects of time for the parameters CMJ (*p* = 0.002, d = 0.55) and SJ (*p* = 0.004, d = 0.51), the Illinois agility test with ball (*p* = 0.016, d = 0.51), and the ball kicking velocity test (*p* = 0.003, d = 0.54). A significant group-by-time interaction was found for the 30-m sprint test (*p* < 0.001, d = 0.76). Post-hoc tests indicated a significantly improved linear sprint speed time for the ES group (*p* = 0.006, d = 0.85, Δ-5%) and a significant performance decline for the SE group (*p* = 0.02, d = 0.75, Δ + 2%) (Fig. 2).

**Table 2:** Group-specific mean values and standard deviations for physical fitness measures from pre to post (*p*-values and effect sizes [Cohen’s d])

Variable	Group SE				Group ES				Main Effect of **Group** (*p*-value, Cohen’s d)	Main Effect of **Time** (*p*-value, Cohen’s d)	Interaction of Group*Time (*p*-value, Cohen’s d)
	PRE	POST	Δ %	95% CI	PRE	POST	Δ %	95% CI			
**Countermovement jump (CMJ)** [cm]	28.6 ± 4.4	30.3 ± 5.9	6%	1.5–4.7	28.7 ± 5.2	30.9 ± 6.0	7%	0.7–3.4	0.84 (0.03)	**0.002 (0.55)**	0.71 (0.06)
**Squat jump (SJ)** [cm]	27.5 ± 4.9	28.6 ± 4.0	4%	1.2–3.3	29.9 ± 5.5	30.3 ± 4.9	1%	0.8-4.0	0.48 (0.11)	**0.004 (0.51)**	0.29 (0.18)
**30-m sprint ** [s]	**4.7 ± 0.3**	**4.8 ± 0.3**	**2%**	0.7–3.3	**4.8 ± 0.4**	**4.5 ± 0.3**	**-5%**	0.6–3.1	0.37 (0.18)	0.12 (0.31)	**< 0.001 (0.76)**
**Illinois agility test** [s]	15.7 ± 0.6	15.7 ± 0.5	0%	3.5–9.4	16.0 ± 0.7	16.0 ± 0.8	0%	2.3–11.5	0.15 (0.28)	0.75 (0.06)	0.95 (0.003)
**Illinois agility test with ball** [s]	20.4 ± 1.1	19.7 ± 1.0	-4%	4.9–13.3	21.1 ± 1.5	20.3 ± 1.1	-4%	2.7–13.2	0.08 (0.36)	**0.016 (0.51)**	0.88 (0.03)
**Ball kicking velocity** [km/h]	90.9 ± 10.8	94.0 ± 6.9	3%	2.3–6.2	90.1 ± 11.4	93.9 ± 6.9	4%	7.1–34.7	0.86 (0.03)	**0.003 (0.54)**	0.78 (0.11)

## Discussion

Here, we examined the intrasession CT sequencing effects on measures of physical fitness and soccer-related performance in adolescent male soccer players. The main findings demonstrate that the applied intrasession CT sequencing schemes (SE vs. ES) resulted in similar changes in most measures of physical fitness (SJ, CMJ) and soccer-related performance (Illinois agility test with ball, ball kicking velocity) in male adolescent soccer players. For linear sprint speed (30-m sprint), ES produced larger performance improvements, while SE indicated a performance decline. Accordingly, our research hypothesis can be partially accepted.

The results of this study are in line with previous research examining CT effects in youth and adult athletes and non-athletes. For instance, Enright and Morton [1] investigated the effects of intrasession CT sequencing (SE vs. ES) and found similar training effects for SJ, CMJ and peak isokinetic torque of the hamstrings and quadriceps muscles in adolescent (17.3 ± 1.6 years) soccer players. The authors further reported larger effect sizes for ES compared with SE for 10-m linear sprint speed, 1-RM half back squat, rate of torque development, and muscle morphology (e.g., ultrasound based muscle thickness, fascicle length, pennation angle), without reaching the level of statistical significance [1].

In another study, Makhlouf et al. [2] examined the effects of intrasession SE vs. ES sequencing vs. SE conducted on alternate days vs. soccer training only on measures of physical fitness and soccer-related performance in adolescent male soccer players aged 13.7 ± 0.5 y. The authors reported significantly greater improvements for linear sprint speed (10-m and 30-m) and 1-RM squat when CT was conducted on the same day (intrasession SE and ES) vs. CT on alternated days. Further, both intrasession CT groups compared to the soccer only group showed significantly greater improvements for measures of physical fitness and soccer-related performance (10-m and 30-m sprint tests, Yo-Yo test, Agility 15-m test, Ball 15-m test). McGawley and Anderson [14] examined the effects of intrasession SE vs. ES sequencing on measures of physical fitness in adult male elite soccer players aged 23 ± 4 y. Significant main time effects but no significant group-by-time interactions were found for selected measures of linear sprint and CoD speed (10-m sprint, 6 × 30-m repeated sprint, and 40-m agility). In a non-athletic population of healthy males aged 32.0 ± 6.5 y, Küüsmaa and Schumann [6] examined the effects of intrasession SE vs. ES sequencing on 1-RM leg-press performance, vastus lateralis cross-sectional area (measured via ultrasound), and endurance performance (all-out aerobic cycling test). The authors observed significant main time effects for all measured outcomes. For endurance (ergometer test), a significant group-by-time interaction was reported in favor of ES. In this study, the strength training regime consisted exclusively of machine-based strength exercises.

In a non-athletic population of prepubescent youth, Alves and colleagues [16] found that intrasession SE resulted in greater medicine ball throw and standing long jump performances compared to intrasession ES. ES showed greater 20-m linear sprint and V02max improvements compared with SE.

In an attempt to summarize the available literature on CT in youth, Gäbler et al. [12] systematically reviewed and meta-analyzed the available studies examining CT effects on measures of physical fitness and athletic performance in youth and youth athletes. The authors identified 15 studies and concluded that CT conducted on the same day had “at worst no interfering but perhaps a potentiating effect” on measures of physical fitness (e.g. exercise economy, CMJ) and athletic performance (e.g., performance in time trials) compared to single-mode endurance or strength training in adolescent athletes aged 11 to 18 y (median = 14.1). Moreover, Gäbler and Granacher [11] postulated that the intrasession CT sequencing (SE vs. ES) effects may vary according to the maturational status and training background. The authors reported distinct adaptive changes for measures of physical fitness (e.g., VO2max, CMJ, 1-RM leg press and squat) in around PHV versus post PHV youth when the two different intrasession sequencing schemes (SE vs. ES) were applied. In the same context, further disparities were also found between adult athletes and non-athletic adults [11]. While primarily adults have exhibited interfering effects, especially for strength and hypertrophy gains within CT compared to single mode endurance or strength training [13, 37], adolescents (10–18 y) seem to show greater resistance against CT-induced interference effects [11]. The underlying mechanisms might be related to several factors. Lower androgen levels in youth and subsequent smaller potential for strength and hypertrophy gains were previously suggested to minimize interference between the two training modalities due to the added endurance stimuli [13, 37]. Young athletes further exhibit less training-induced fatigue due to faster recovery periods from training which may also contribute to minimized interference through CT [38]. In terms of sequencing effects induced by intrasession SE vs. ES, Coffey et al. [13] hypothesized that the interference is likely a consequence of antagonistic intracellular signaling mechanisms between mTOR (main mediator for strength and hypertrophy training adaptations), AMPK (driving mitochondrial biogenesis following endurance training), as well as peroxisome proliferator activated receptor gamma coactivator-1 alpha (PGC-1α [inducing angiogenesis as a response of endurance training]) [5, 13]. When hypertrophy and endurance training are performed in close temporal proximity, primarily PGC-1α has been subjected to block the mTOR pathway, but not vice versa [13]. This may be the physiological basis for the intrasession sequencing effect between SE vs. ES in regards to altered sprinting adaptations in linear sprint speed, as measured in our and other studies across youth and adult populations [1, 6, 12, 14]. It is important to mention that our strength training protocol consisted mainly of plyometric exercises known to improve activities conducted in the stretch shortening cycle through neuromuscular adaptations. The mechanisms of interference with CT for these adaptations have previously been proposed to derive from altered neural recruitment, neuromuscular fatigue and the inability to develop adequate force due to the added endurance training stimuli [39].

Coffey and colleagues further postulated that the underlying effect is greater in adults (possibly due to larger potential for hypertrophy and hence larger interference potential [11, 37]) and more pronounced in athletic populations compared to untrained individuals [13]. The authors specify that highly trained athletes require greater and more specific training loads to disrupt homeostasis and promote further adaptation. Therefore, any added stimulus (through CT) that is not directed towards the final adaptational goal (strength or endurance) is more likely to result in impaired progress. With higher training loads and specificity of desired training outcomes, the potential for an interference effect, especially for strength and hypertrophy gains, is exacerbated [13].

Next to the metabolic and neuromuscular reasoning for interference effects, it is also vital to consider acute implications of closely sequenced training sessions. Residual fatigue from prior training sessions within intrasession CT can influence the following session by depleted muscle glycogen levels, peripheral muscular fatigue and altered hormonal levels.

These phenomena are known as the acute hypothesis of CT interference effects [40]. It remains unclear how different sequencing schemes (microcycle, same day, intrasession) in comparison to each other can affect these beneficial / interfering effects in athletic youth populations.

### Study Limitations and Future Directions

This study is not without methodological limitations that should be acknowledged.

First, dropouts due to participants missing exercise sessions introduce potential bias and limit the robustness of conclusions. The missed training sessions were likely due to the in-season period which coincides with winter and early spring time in the Netherlands. Hence, illness-induced absence from training was rather high. Although steps were taken to address attendance discrepancies (exclusion of all participants with less than 50% overall adherence), the potential impact on the results remains a study limitation as inconsistent adherence can further disrupt the uniformity of the training stimulus, confound the observed outcomes and compromise the magnitude of potential training sequencing effects. We therefore assessed adherence patterns and found no significant differences between the SE (69 ± 14%) and ES (66 ± 11%) group (Table 1). Second, it is important to mention that individual differences within the study cohort may have an impact on the results of this study. However, the two experimental groups were rather homogeneous since we could not detect any baseline between group differences for anthropometric and demographic measures (e.g., age, body height, body mass, soccer experience, age from PHV, age at PHV) (Table 1). Future investigations should additionally consider taking body composition measures such as lean mass and body fat percentage in order to ensure better subject homogeneity. Further, it is important to point out that this study tested two different intrasession CT sequencing schemes (SE vs. ES), without a control group performing the strength and endurance training on alternate days. Hence, these results cannot elucidate whether CT conducted in the same training session produced better results than CT on alternate days, as reported in the aforementioned literature [2]. To gain a greater understanding of the underlying interference and sequencing effects, further research investigating the driving physiological mechanisms should be performed.

The likely small effects of training sequencing require large sample sizes. This offers major feasibility challenges, that have contributed to the current paucity of available data. In order to better understand the true effects in the niche of young athletes, it is crucial to aggregate the data from this and similar (future) studies. Future research should directly compare the different CT sequencing modalities (microcycle, same day, intrasession) as well as the type of included strength (e.g., weight lifting, plyometrics) and endurance training (e.g., high intensity interval training) in order to determine the most effective CT approach in adolescent soccer players.

## Conclusions

CT seems to be a suitable and effective exercise program to develop measures of physical fitness as well as soccer-related performance in adolescent male soccer players. Our data suggest that youth soccer coaches can choose either SE or ES intrasession sequencing of CT during in-season training and expect similar improvements in most measures of physical fitness and soccer performance. If the goal is to improve linear sprint speed in adolescent soccer players, intrasession ES sequencing should be prioritized over SE. Soccer coaches can apply these findings when designing soccer training protocols for adolescent male players.

### Electronic Supplementary Material

Below is the link to the electronic supplementary material.

Supplementary Material 1

## Data Availability

The datasets generated and analyzed during the current study are not publicly available due to participants data protection policies but are available from the corresponding author on reasonable request.

## Abbreviations

AMPK: Adenosine monophosphate activated protein kinase
CMJ: Countermovement jump
CT: Concurrent Training
ES: Endurance-strength
ICC: Intra-class correlation coefficients
mTor: mechanistic target of rapamycin
PGC-1α: Peroxisome proliferator activated receptor gamma coactivator-1 alpha
PHV: Peak-height-velocity
1-RM: One-repetition maximum
SE: Strength-endurance
SJ: Squat jump
Y: Years
