# Supplementary material for: Sequencing Effects of Concurrent Strength and Endurance Training on Selected Measures of Physical Fitness in Young Male Soccer Players: A Randomized Matched-Pairs Trial
Source: Sports Med Open. 2024 May 23;10:62. doi: 10.1186/s40798-024-00726-4 (PMC11116357; doi:10.1186/s40798-024-00726-4)
Supplement: Supplementary file 1 — Supplementary Material 1 [file 40798_2024_726_MOESM1_ESM.docx]

**Supplementary Information**

**Study title:**

“Sequencing effects of concurrent strength and endurance training on selected measures of physical fitness in young male soccer players: A randomized matched-pairs trial”

**Authors:**

R. Blechschmied, M. Hermse, M. Gäbler, M. Elferink-Gemser, T. Hortobagyi and U. Granacher

**For publication in:**

Sports Medicine -Open in “Original Research Articles”

**Corresponding Author:**

Prof. Urs Granacher, PhD

Email: urs.granacher@sport.uni-freiburg.de

**Competing interest:**

The authors Roland Blechschmied, Matthijs Hermse, Martijn Gäbler, Marije Elferink-Gemser, Tibor Hortobágyi and Urs Granacher declare that they have no conflicts of interest to disclose related to this research.

**Funding:**This article is part of the research project “Resistance Training in Youth Athletes” that was funded by the German Federal Institute of Sport Science (ZMVI1-081901 14-18 and ZMVI4-081901/20-23). The funders had no role in study design, data collection and analysis, decision to publish, or preparation of the manuscript.

**Supplement 1:** Exercise protocols for the two interventions.

|  | **Session 1** |  |  |  |  |  |  | **Session 2** |  |  |  |  |  |  |
| --- | --- | --- | --- | --- | --- | --- | --- | --- | --- | --- | --- | --- | --- | --- |
|  | Strength |  | | Intermittent endurance | | | | Strength |  | | Intermittent endurance | | | |
| Week | Exercise | Sets | Reps/ Time/ Distance | Bouts | Active (min) | Rest (min) | Avg Time > 80% HRmax | Exercise | Sets | Reps/ Time/ Distance | Bouts | Active (min) | Rest (min) | Avg Time > 80% HRmax |
| 1 | Squat jump Prone Bridge Lateral jump Floor bridge on ball | 3 3 3L & 3R 3 | 10 10 10 10 | 3 | 3 | 1 | N/A | CMJ Prone partner ball toss Lunges Side plank | 3 3 3 3L & 3R | 15 10 15 30 s | 3 | 3 | 1 | NA |
| 2 | No training | | | | | | | Tuck jump Mountain climbers Hordes Hip abduction | 3 3 3 3 | 15 30s 5 x 5 10 | 3 | 3 | 1 | 08:25 |
| 3 | Lunges Planking Single leg jump Nordic hamstring | 3 3 3L & 3R 3 | 15 30s 20 8 | 3 | 3 | 1 | 04:05 | CMJ + sprint Side rotation with ball  High hordes Shoulder duel | 3 3 3 4 | 13 + 3 16 5 x 5 2 | 4 | 2 | 1 | 05:34 |
| 4 | Single leg squat jump Side plank Forward bounding Leg curl | 4 4 3 4 | 10 20s 4 13 | 3 | 3 | 1 | 07:02 | Squat jump Back with ball Hordes Side step with band | 3 3 3 3 | 10 10 3 25 s | 3 | 3 | 1 | 10:55 |
| 5 | Single leg jump Plank Partner carrying Reversed plank | 3 3 3 3 | 20 30s 8 10 | 4 | 2 | 0.5 | 06:09 | Hurdles Band jump CMJ + sprint Mountain climbers | 3 2 3 3 | 30 16 9 + 3 30 s | 3 | 3 | 1 | 09:22 |
| 6 | Band resisted sprint with partner Side bridge Single leg squat Nordic hamstring | 4 4 4 6 | 30 m 40 s 8 20 s | 2 | 6.5 | 1 | 07:01 | Lateral leg raise Jump lunges Heel touch Drop jump | 6 3 3 3 | 12 15 30 s 15 | 3 | 4 | 1 | 08:50 |
| 7 | Squat jump + CoD Plank Speed skater lunges Nordic hamstring | 4 3 4 4 | 4 40 s 14 5 | 3 | 3.5 | 0.5 | 07:40 | Fence squat Cart leg drop Burpees (no push-up) Single leg jump race | 3 3 3 3L & 3R | 30s 20 10 40m | 3 | 3,5 | 0,5 | 08:57 |
| 8 | No training | | | | | | | Drop jump Crunches Partner carry squat Band resisted leg abduction | 3 3 3 3L & 3R | 8 40s 8 12 | 3 | 3 | 1 | 08:13 |
| 9 | Single leg squat Nordic hamstring Pistol squat/single leg jump | 3L & 3R 3 3L & 3R | 8 5 5 | 1 | 9.5 | 0 | 06:38 | Circuit inclusively: Hurdles Single leg jumps CMJ + 1 sprint | 3 a round a round a round | 4 rounds 5 9 5 + 1 | 3 | 3 | 0,5 | 07:51 |
| 10 | Single leg jump forward Side bridge + 1 leg up Pistol squat Nordic hamstring | 3L & 3R 3L & 3R 3L & 3R 3 | 8 20 8 5 | 2 | 5.5 | 1 | 09:00 | Drop jump CMJ + CoD Shoulder duel | 3 3 3 | 30 s 35 s 40 s | 3 | 3,5 | 0,5 | 08:04 |
| 11 | Partner carry squat Nordic hamstring Forward moving CMJ Plank | 3 3 3 3 | 5 5 7 30s | 2 | 4 | 1 | 04:03 | CMJ + CoD Lateral hurdles Jumping lunges | 3 4 3 | 10 + 3 16 12 | 3 | 3 | 0,5 | 08:47 |
| 12 | Single leg jump forward Side bridge Pistol squat Nordic hamstring | 3L & 3R 3L & 3R 3L & 3R 3 | 8 20 8 5 | 2 | 5 | 0.5 | 07:39 | No training | | | | | | |
